# Smart drug delivery and responsive microneedles for wound healing

**DOI:** 10.1016/j.mtbio.2024.101321

**Published:** 2024-10-29

**Authors:** Meixuan Liu, Jing Jiang, Yiran Wang, Huan Liu, Yiping Lu, Xingang Wang

**Affiliations:** a Department of Burns & Wound Care Center, The Second Affiliated Hospital, Zhejiang University School of Medicine, Hangzhou, 310009, China; b Senior once Class 5, Shanghai Pinghe School, Shanghai, 200000, China

**Keywords:** Microneedles, Wound healing, Delivering drug system, Smart responsive microneedles

## Abstract

Wound healing is an ongoing concern for the medical community. The limitations of traditional dressings are being addressed by materials and manufacturing technology. Microneedles (MNs) are a novel type of drug delivery system that has been widely used in cancer therapy, dermatological treatment, and insulin and vaccine delivery. MNs locally penetrate necrotic tissue, eschar, biofilm and epidermis into deep tissues, avoiding the possibility of drug dilution and degradation and greatly improving administration efficiency with less pain. MNs represent a new direction for wound treatment and transdermal delivery. In this study, we summarise the skin wound healing process and the mechanical stimulation of MNs in the context of the wound healing process. We also introduce the structural design and manufacture of MNs. Subsequently, MNs are categorised according to the loaded drugs, where the design of the MNs according to the traumatic biological/biochemical microenvironment (pH, glucose, and bacteria) and the physical microenvironment (temperature, light, and ultrasound) is emphasised. Finally, the advantages of MNs are compared with traditional drug delivery systems and their prospects are discussed.

## Graphical abstract

**Figure:**
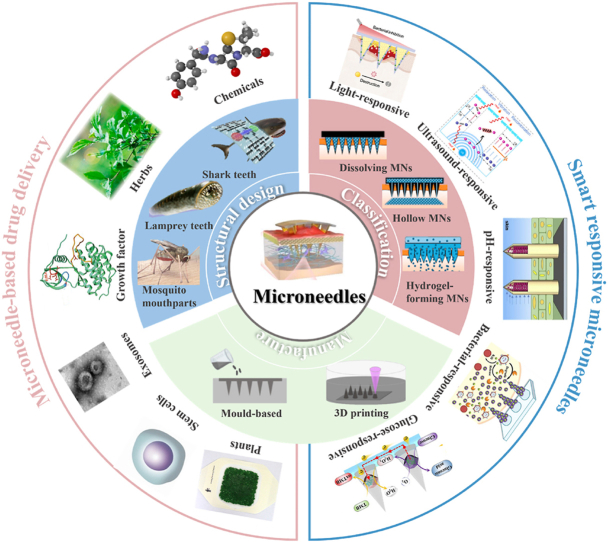

## 1 Introduction

The skin is the human body's largest organ, and it is a natural barrier that protects the body from invasion by microorganisms, prevents the loss of water, electrolytes and nutrients, and maintains the stability of the internal environment [1]. Once the skin is damaged by trauma or burns, its functions weaken or are lost, which endangers health. Therefore, accelerating wound healing has been a concern for the entire medical field. Various drug delivery systems have emerged to overcome the limitation of conventional dressings of only delivering the loaded drug to the skin surface. Microneedles (MNs) are an emerging drug delivery system developed in 1976 and first used in 1998 [2]. MNs are now widely used in the biomedical field due to their minimal invasive, convenience, local controllable administration, and personalised design, particularly for cancer treatment [3], dermatological therapy [4], and insulin [5] and vaccine [6] delivery.

MNs are composed of multiple micron-sized fine tips attached to a base in an array. MNs penetrate the stratum corneum, a barrier that impedes drug permeation, thereby producing micro-sized mechanical channels within the epidermis and superficial dermis. MNs also penetrate wound clots and scar tissue to deliver drugs to the wound bed, where they exert pharmacological effects. The design of MNs is customizable, with parameters such as length, size, shape, and loaded drugs to the specific therapeutic application. Discomfort, like pain, is minimized or negated by carefully controlling the depth to avoid stimulating the sensory nerves and dermal capillaries. MNs provide new possibilities for personalised and customised treatment.

In recent years, smart responsive MNs have been rapidly developed and widely used in the drug delivery system. These innovative MNs platforms, embedded with drug-loaded responsive biomaterials, are designed to sense and respond to the surrounding environment, enabling real-time feedback and controlled release of therapeutic agents in response to physiological or biochemical cues such as temperature, light, ultrasound, pH, glucose and bacteria [7,8]. Smart responsive MNs could break the limitations of low efficient delivery, simplistic delivery mechanisms, and inability to adapt to individualised requirements, which promise to emerge as a groundbreaking tendency in the realm of biomedical engineering and biomanufacturing.

Previous literature related to MNs have mostly focused on the manufacture of MNs and categorised them based on their morphology. Beyond providing a comprehensive synopsis of the historical development, structural designs and fabrication techniques of MNs, this review featured the classification of MNs based on the loaded drugs. Finally, the review delved into an extensive examination of smart responsive designs of MNs, as well as their applications in wound healing.

## 2 History of microneedles

The concept of microneedles (MNs) was initially introduced by Alan Richard Wagner in 1958, who envisioned a technology smaller than conventional hypodermic needles to relieve pain. In 1976, Gerstel and Place first proposed the application of MNs as transdermal drug delivery in their article titled “Drug delivery device”. However, due to unsophisticated scientific techniques, they and other scientists did not fabricate MNs loaded with drugs during the next 20 years [9]. In 1998, Herry et al. first applied MNs for transdermal drug delivery. They used an ion etching technique to fabricate silicon MNs loaded with calcein [2]. Since then, scientists have been delivering various drugs using MNs, and MN research and development have gone into overdrive. The material and manufacturing techniques for MNs have experienced many iterative updates. According to Ingrole's survey, from 1990 to 2018 there were 1027 original research articles, including 73 % related to molecule delivery and 13 % related to disease diagnosis. The delivery of various molecules, including small molecules, biomolecules, and vaccines, emerged as a predominant area of study, with dissolving and coated MN designs being the most prevalent [10].

Nonetheless, the limitation of manufacturing technologies impeded the production and commercialization of MNs. Most applications for MNs were in the laboratory rather than being translated into clinical practice. With the development of technology and materials, the first approved MN product was the derma roller, which was a cylindrical roller with solid MNs to treat scars and hyperpigmentation. The 3M Company produced hollow MN patches to deliver biologics and other small molecules for clinical treatment by transdermal drug administration [11]. These clinically applied MNs have simple structures and poor drug-loading capacity, with limited application value. In the future, MNs will require modification and refinement to enhance their efficacy in drug delivery and to realize smart responsiveness. This review focuses on the design and application of MNs for wound healing management.

## 3 Phases of wound healing and microneedle mechanical functions

Wound healing is a complex repair process that is generally divided into four overlapping phases: coagulation and haemostasis, inflammation, proliferation, and remodelling [12]. Following skin injury, platelets, a rapid response is initiated with platelets, fibrin, and red blood cells collaborating to form clots that occlude the wound bed. Serotonin, histamine and bioactive factors are released, which increase vascular permeability to recruit inflammatory cells to the wound bed. At the same time, keratinocytes, fibroblasts and endothelial cells undergo migration, proliferation, and differentiation to effect re-epithelialisation, fibrous hyperplasia, and angiogenesis. Finally, wound healing enters the maturation and remodelling phase. The fibroblast-collagen matrix is remodelled, the extracellular matrix (ECM) is organised, and the wound contracts to restore the normal appearance and function of the injured site [13].

**Fig. 1:**
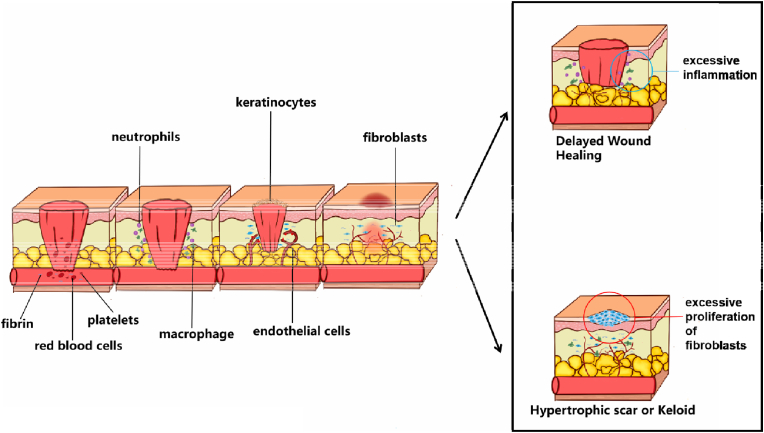
Phases of wound healing.

Some pathophysiological and metabolic disorders alter the normal wound healing process, leading to delayed wound healing such as diabetic ulcers. Causes of delayed wound healing in diabetic ulcers include chronic inflammation, impaired neovascularisation, decreased collagen synthesis, increased protease levels, and impaired macrophage function [14]. In addition, if the proliferative phase is inappropriately prolonged, excessive fibroblast proliferation and collagen deposition will form a hypertrophic scar or keloid, representing an adverse outcome of wound healing [15] (Fig. 1).

**Fig. 2:**
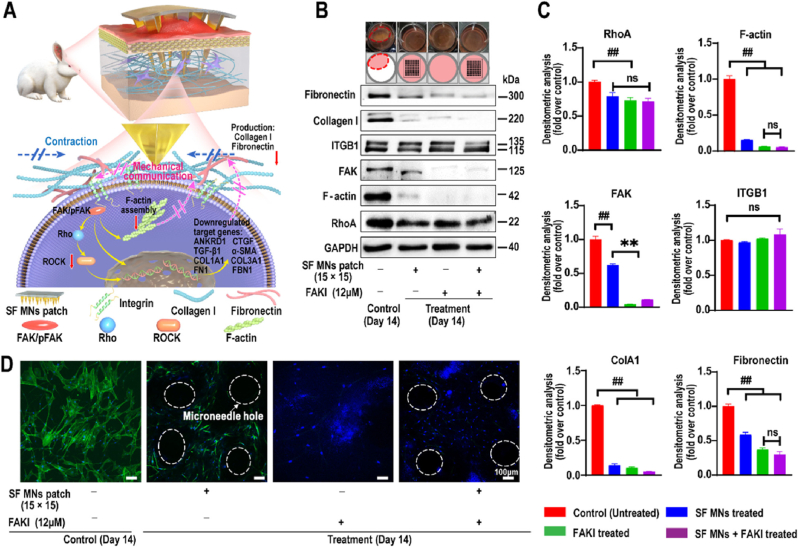
SF MNs alter the mechanical communication between fibroblasts and the surrounding matrix and result in a cascade of cellular responses. (A) mechanism diagram of SF MNs. (B) and (C) Western blotting of integrin, FAK, RhoA, F-actin, type I collagen and fibronectin involved in fibroblasts with the treatment of FAKI, SF MNs, and FAKI + SF MNs. (D) CLSM of the intracellular F-actin meshwork of fibroblasts under different treatments. Reproduced with permission [18]. Copyright 2022, ACS Nano.

MNs exhibit electric field stimulation and mechanical force functions. A potential difference of approximately −23.4 mV exists between the stratum corneum and the dermis. When a single needle penetrates the skin, the Na/K-pump becomes activated, producing transient charging and discharging. For microneedle patches with hundreds of needles per square centimetre, the cells adjacent to the needles are repeatedly stimulated by penetration, leading to a permanent activation and the generation of a polarized electromagnetic field (EMF). The EMF could upregulate the expression of DNA associated with proliferation in surrounding cells, thereby increasing the secretion of growth factors for wound healing [16]. MNs demonstrated favorable mechanical properties to puncture the skin. They cause minor controllable injuries that disrupt collagen chains at the base of the dermis, and induce a natural wound healing cascade that promotes neovascularisation and neocollagenesis [17]. Qing et al. used silk fibroin MN patches in a rabbit ear hypertrophic scar model. The MNs interrupted the mechanical communication between fibroblasts and the ECM, and promoted ECM remodelling via the integrin-Fak signalling pathway to reduce the secretion of collagen Ⅰ and fibronectin [18] (Fig. 2). The MN-mediated mechanotherapy contributed to improve the aesthetic and mechanical characteristics of scars. Although MNs themselves have electric field stimulation and mechanical forces to promote wound healing, the treatment of chronic wounds is impeded by many complex factors that inhibit the healing process. The efficacy of MNs used in isolation for treating chronic wounds is limited. Consequently, MNs are frequently loaded with various drugs to endow them with anti-inflammatory, antibacterial, angiogenic and anti-scar properties.

## 4 Structural design and manufacture of microneedles

### 4.1 Microneedle classification

The manufacture of MNs has undergone rapid development. Traditional MNs are fabricated from silicon, metals, ceramics, and other materials. However, these have been progressively phased out because of their propensity for fracturing, potential for environmental contamination, and associated biohazards. Currently, polymer MNs with robust biocompatibility are primarily used, such as carboxymethylcellulose (CMC), polyvinyl pyrrolidone (PVP), polyvinyl alcohol (PVA), poly (lactic-co-glycolic acid) (PLGA), and methacryloyl hyaluronic acid (HA) [19]. Polysaccharides derived from animal and plant sources, such as HA, glucan, chitosan (CS), cellulose, and sodium alginate, have gained popularity because of their biocompatibility, biodegradability, and environmental friendliness [20].

In addition, the metal-organic frameworks (MOFs) are an emerging synthetic material with great promise in drug delivery. MOFs are porous structures formed by the coordination of metal ions and organic ligands. MOFs exhibit properties of ultra-high porosity, large specific surface area, and high thermal conductivity, rendering them an excellent host matrix for encapsulating drugs. They hold the potential to store and release metal ions, such as Zn-MOF and Mg-MOF [21]. Zinc ions released from Zn-MOF could kill bacteria by destroying bacteria capsules and stimulating the production of reactive oxygen species (ROS).

**Fig. 3:**
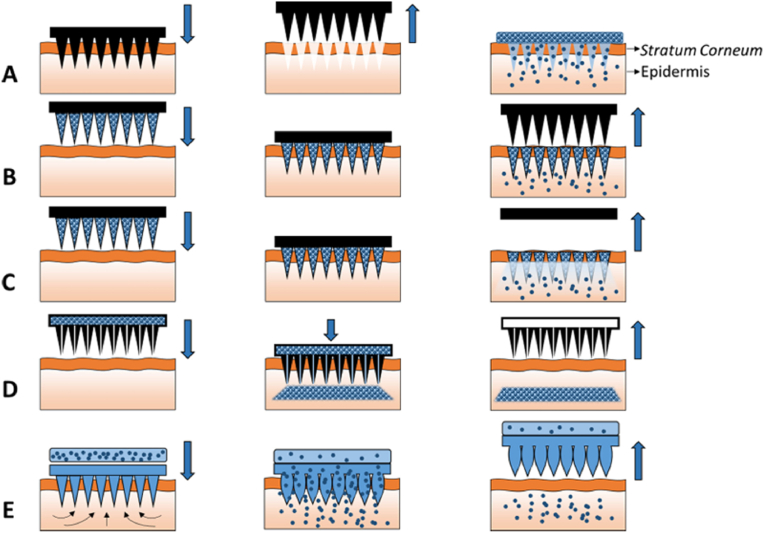
Schematic representation of five types of microneedle administration methods. (A) Solid MNs. (B) Coated MNs. (C) Dissolving MNs. (D) Hollow MNs. (E) Hydrogel-forming MNs. Reproduced under terms of the CC-BY license [9]. Copyright 2016, Materials Science and Engineering: R. Reports.

MNs have been categorised into five types based on their loading methodologies (Fig. 3) [3]. 1) Solid MNs are directly cut from solid materials (e.g. silicon and titanium dioxide) and easily inserted into the skin with high hardness. They are not loaded with drugs, but rather puncture the epidermis to form microchannels. 2) Coated MNs are fabricated by depositing drug and polymer coatings via techniques including spray drying [22], inkjet printing [23], dip coating [24], and electrochemical deposition [25]. They transport drugs attached to the surface deep into the dermis, and the MN patch is then removed for reuse [26]. 3) Dissolving MNs are prepared from biocompatible water-soluble materials such as PVA, PLGA, and HA [6,27,28]. The encapsulated drugs are naturally released as the MN degrades, which eliminates the need to remove the needle. 4) Hollow MNs feature an internal cavity and a bore at the needle tip, functioning akin to a microsyringe for directly injecting drugs through MN holes [29]. They have the largest drug-delivering capacity due to their hollow structure. The close arrangement of dermal fibers may occlude the needle hole upon insertion. 5) Hydrogel-forming MNs are constructed from soluble hydrophilic cross-linked polymers. When inserted into the skin, hydrogel-forming MNs absorb matrix water and expand. Based on the crosslinking strength of the hydrogel network, the controlled release of drugs is achieved [30].

### 4.2 Microneedle manufacture

MN manufacturing techniques vary, and different types of MNs require different manufacturing techniques. The most commonly used manufacturing technique is based on a mould. First, the desired MN moulds are fabricated based on previous designs using techniques such as laser cutting, laser ablation, photolithography, etching, micro-stereolithography, and three-dimensional (3D) printing. Subsequently, curable materials are poured into the mould to form a negative mould. The final MN can be obtained by filling the chosen material into the negative mould using vacuum, followed by centrifugation, imprinting, and spin coating [31]. Mould-based fabrication not only has the advantages of reusability and high accuracy, but also enables the production of double or multiple-layer MNs to meet different needs. Double-layered MNs achieve tip-to-base separation to allow precise hierarchical drug delivery at different phases of wound healing. For example, loaded antibiotics or haemostatic drugs in the base play an antibacterial and haemostatic role during the early phase, and loaded growth factors in the tip are slowly released during the proliferative and remodelling phases [32,33]. Another less-used method is drawing lithography [34]. Drawing lithography involves placing a polymer on a high-temperature substrate, stretching it with a pipette, and waiting for it to cool or light-crosslink to solidify the MNs, which are mostly used to manufacture glass MNs. Furthermore, 3D printing technology can be used to directly print MNs without the need for a mould, using MN materials as ink and designing with computer-aided design software, thereby achieving highly accurate industrial production [35].

### 4.3 Microneedle structural design

The structure of the MN is designed to penetrate the stratum corneum into the epidermis and to avoid nerve and blood vessels in the dermis without discomfort [36]. The epidermis is generally 50–100 μm thick, and that on the palms and soles is 1500 μm thick. Therefore, MNs are designed to range from 150 to 1500 μm in length, with bottom diameters of 50–250 μm and tip diameters of 1–25 μm [37]. In addition to penetrating the epidermis, MNs must penetrate bacterial biofilms, which are prevalent in chronic wounds. For example, typical mature *Staphylococcus aureus* biofilms can be 150 μm thick above the wound surface and invade 190 μm below the wound surface [38]. The depth of the biofilm should be considered when designing an antimicrobial MN.

**Fig. 4:**
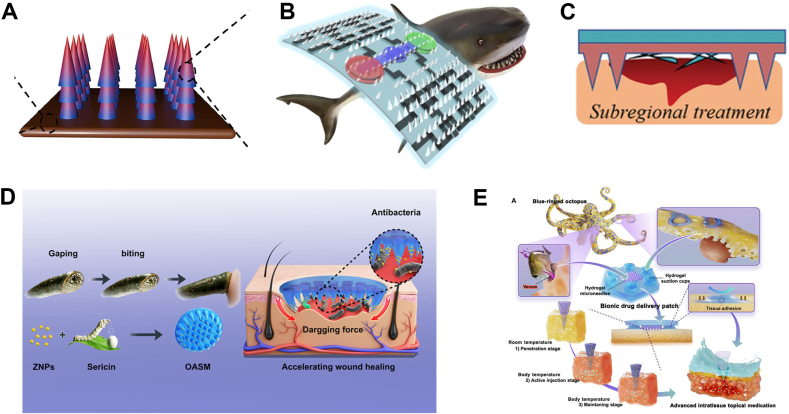
The simulated design of microneedles. (A) Porcupine quill barbs. Reproduced with permission [41]. Copyright 2023, Acta Biomaterialia. (B) Shark teeth. Reproduced with permission [42]. Copyright 2023, Acta Biomaterialia. (C) Mosquito mouthparts. Reproduced with permission [43]. Copyright 2023, Advanced Healthcare Materials (D) Lamprey teeth. Reproduced with permission [44]. Copyright 2022, Nano Letters). (E) Blue-ringed octopus. Reproduced under terms of the CC-BY-NC license [45]. Copyright 2023, Science Advances.

The morphology of the MN affects skin penetration and drug delivery efficacy. A conical shape is more resistant to compression than a spear or arrow in the longitudinal axis, allowing for insertion into the skin with minimal pain. Stress-strain analysis revealed that the indentation performance was improved by increasing the number of vertices on the MN base morphology, which produced deeper perforation sites and greater drug penetration. For example, MNs with a star-like base exhibited superior skin penetration capability compared to those with circular, triangular, or square bases [39,40]. Scientists have drawn inspiration from natural organisms to simulate MN designs that improve adhesion and penetration. Tianqi et al. designed an MN to enhance adhesive strength based on the tiny backward barbs of a porcupine quill (Fig. 4a) [41]. Maoze et al. were inspired by shark teeth to design an MN patch featuring flat and inclined structures (Fig. 4b) [42]. Wang et al. designed rigid and long MNs to fix the skin, coupled with shorter MNs for insertion into the wound bed. Their design inspired by mosquito mouthparts to reduce irritation in sensitive non-healing wounds (Fig. 4c) [43]. Yan et al. was inspired by lamprey teeth to design a circular base, and the MN tip tilted inward from the edge layer by layer to provide the oriented dragging force (Fig. 4d) [44]. Inspired by the predation mechanism of the blue-ringed octopus, Zhu et al. fabricated hydrogel suckers around MNs to provide wet bonding (Fig. 4e) [45].

The inter-needle spacing within MN arrays affects the penetration force (PF; the reaction force preceding the characteristic drop following skin puncture), penetration efficiency (PE; percentage of needles on an array successfully penetrating the skin), and skin strain. However, diametrically opposite results have been reported by different studies. Kochhar et al. observed an increase in PF and PE with an increase in MN spacing from 0.4 to 2.4 mm [46]. Olatunji et al. reported a decrease in PE with increasing MN spacing [47]. Shu et al. demonstrated that PE increased, and PF decreased, as MN spacing was increased between 0.156 and 1.75 mm [48]. Matthew et al. showed that both PF and skin strain increased with increased MN spacing, with an optimal spacing of 2–3 mm and essentially stable forces beyond 3 mm. As MN spacing increased, the overlap of the deformation zones below each needle decreased, as did the stretch caused by the indentation effect of adjacent needles, thereby increasing PF. Furthermore, they used the same spacing in the two- and nine-needle models, and the number of MN tips had no effect on PF or skin strain [49].

## 5 Microneedle-based drug delivery for wound healing

### 5.1 Delivering-chemical MNs

#### 5.1.1 Antimicrobial agents

Bacterial infection is one of the main reasons for poor or delayed wound healing. Biofilms are present in 75 % of chronic wound cases [50]. Systemic administration of antibiotics (e.g. intravenous administration) can lead to drug resistance and side effects, such as phlebitis, renal damage, and hepatic impairment. Topical application of antibiotics effectively reduces these complications. Thus, antibiotics were the first loaded drugs to be emphasised by researchers, including vancomycin (VAN) [51], ceftriaxone [52], tetracycline [32,53], doxycycline (DOX) [27], moxifloxacin [28], levofloxacin [54], macrolides (erythromycin and azithromycin) [55], and chloramphenicol [56] (Table 1).

**Table 1:** Summary of MNs of antimicrobial and anti-scar agents in wounding healing and scar inhibiting.

Category	Delivery substance	MNs shape	Materials	Functions	Ref
Antibiotics	Vancomycin	quadrangular pyramidal	tips: PVA; base: PMMA	reduce MRSA growth both *in vitro* and *ex vivo* on skin	[51]
	Ceftriaxone	/	PMVA、Kollicoat®	promote diabetic wound healing	[52]
	rh-EGF (tips) + Tetracycline (base)	quadrangular pyramidal	tips: Gel-CMC; base: HA	promote diabetic wound healing, eliminate bacteria, regulate inflammation and oxidative stress, and promote angiogenesis and tissue regeneration	[32]
	Tetracycline (tips) + Deferoxamine (base)	pyramidal	tips: HA; base: chitosan and silk fibroin	promote diabetic wound healing, reduce inflammatory responses, promote angiogenesis, and facilitate collagen deposition	[53]
	Doxycycline	pyramidal	PLGA, PCL, chitosan	antibiofilm of *Staphylococcus aureus* and *Pseudomonas aeruginosa*	[27]
	Moxifloxacin, lidocaine (LH), and thrombin (TH)	cone-shaped	chitosan, fucoidan, pullulan hydrogel	rapid hemostasis/analgesia and sustained bactericidal action	[28]
	Levofloxacin	pyramidal	PVA; polydopamine nanoparticles; α-amylase	antibiofilm of *Staphylococcus aureus* and *Pseudomonas aeruginosa*	[54]
	Macrolides (erythromycin and azithromycin)	square pyramidal	HA; PVP	enhance wound healing, anti-bacterial	[55]
	Chloramphenicol	pyramidal	gelatin nanoparticles	antibiofilm, especially of *Vibrio vulnificus*	[56]
Antimicrobial peptides	W379	pyramidal	PVP	antibiofilm of *Staphylococcus aureus* and *Pseudomonas aeruginosa*	[57]
	W379	spindle pyramid	PVP; 1-tetradecanol; IR780	photothermal response antibacterial	[58]
Steroids	Triamcinolone acetonide	pyramidal	HA	reduce the volume of keloids	[61]
	Triamcinolone acetonide	cylindrical supporting part and conical tips	HA, hydroxypropyl-β-cyclodextrin	reduce the value of the scar elevation index, downregulation of mRNA expressions of Collagen I and TGF-β1	[62]
	Protocatechuic aldehyde	pyramidal	HA, or HA/gelatin	induce apoptosis, reduce collagen deposition in HSF, and attenuate VEGF-stimulated angiogenesis of HUVECs	[63]
	Betamethasone	pyramidal	HA	painlessly inhibite scar	[64]
Antineoplastic	5-fluorouracil (tips) + Triamcinolone acetonide (base)	square prism and pyramidal tip	tips: chitosan and dextran; base: HA and hydroxypropyl-beta-cyclodextrin	reduce the value of the scar elevation index, downregulation of mRNA expressions of Collagen I and TGF-β1	[65]
	Bleomycin	spindle pyramid	HA	inhibit the proliferation of human hypertrophic scar fibroblasts (hHSFs) and the secretion of TGF-β1	[66]

∗ PVA: polyvinyl alcohol; PMMA: polymethylmethacrylate; MRSA: methicillin‐resistant Staphylococcus aureus; PMVA: Poly (Methyl vinyl ether-alt-maleic acid); Gel-CMC: gelatin carboxymethyl chitosan; HA: hyaluronic acid; PALG: poly(lactic-co-glycolic acid); PCL: poly (Ɛ-caprolactone); PVP: Polyvinyl pyrollidone; VEGF: vascular endothelial growth factor; HUVECs: Human umbilical vein endothelia.

To improve the poor absorption of skin tissue, Ziesmer et al. prepared VAN-loaded MN arrays, which could penetrate through the skin dermis and loaded VAN dose retained within 24 h. The VAN-loaded MNs effectively inhibited *S. aureus* growth *in vivo* and *in vitro* [52]. Gao et al. designed a double-layer loaded MN with the antimicrobial agent tetracycline hydrochloride (TCH) in the tip and the angiogenic drug deferoxamine (DFO) in the substrate, which accelerated rat diabetic wound healing, antibacterial and angiogenesis [53]. Arshad et al. fabricated polymeric MNs loaded with macrolides (erythromycin and azithromycin). The results showed that the azithromycin-loaded MNs were more effective than topical erythromycin for treating *S. aureus* [55]*.* Antimicrobial peptides, which are basic peptides with the same antibacterial function, have been incorporated into MN systems, which contributed to tackle the problem of drug resistance after antibiotic abuse. Xie et al. designed the W379 antimicrobial peptide, comprised of only eight amino acids, which was effective against *Pseudomonas aeruginosa* and methicillin‐resistant *Staphylococcus aureus* (MRSA) *in vivo* (in diabetic defective mice) and *in vitro* (in isolated skin tissues) [57,58].

#### 5.1.2 Anti-scar agents

Local steroid injections and steroid tape/plaster are common clinical treatments for hypertrophic scars and keloids. Steroid therapy reduce inflammation, inhibit fibroblasts, and decrease collagen synthesis. The efficacy of steroid injections alone is variable, with rates of scar regression ranging from 50 % to complete resolution. Recurrence of scars is common upon discontinuation of treatment, with recurrence rates respectively reaching up to 33 % and 50 % after 1 and 5 years [59]. The reason for this phenomenon may be attributed to the local thickened dermis and dense collagen fibres within the scar, which limits the diffusion of the drug and prevents it from maintaining a high concentration at the injection site. Moreover, multiple repeated intralesional steroid injections could lead to intolerable pain, potentially complicating treatment adherence [60]. The structural characteristics of MNs precisely address these shortcomings.

Researchers have designed MNs loaded with triamcinolone acetonide [61,62], protocatechuic aldehyde (PA) [63], and betamethasone [64] to inhibit scars with less pain *in vivo*. In addition, antineoplastic drugs have been used to treat scars. 5-Fluorouracil-loaded MNs inhibited the expression of collagen Ⅰ and transforming growth factor 1β (TGF-1β), thereby reducing collagen fibre deposition and abnormal proliferation of fibroblasts [65]. Dissolving HA-MNs loaded with bleomycin inhibited the proliferation of hypertrophic scar fibroblasts and the secretion of TGF-1β [66]. Interferon is a cytokine with antiproliferative, antifibrotic and antiviral effects [67]. Interferon has been demonstrated to elevate collagenase levels to interfere with collagen synthesis, as well as modulate TCF-β1 to inhibit fibroblast proliferation [68,69]. Interferon has been administered as an adjuvant therapy following the surgical excision of hypertrophic scars or keloids [70]. Wang et al. fabricated carboxymethyl cellulose (CMC) MNs loaded with rhIFNα-1b, facilitating the slow release of interferon through the degradation of CMC. Their experimental results indicated that interferon MNs significantly inhibited abnormal fibroblast proliferation and excessive deposition of collagen fibres, which improved the colour and thickness of the scar tissue [71].

#### 5.1.3 Herbs and their biological extracts

Herbs and their biological extracts hold promise for promoting wound healing, angiogenesis, and collagen deposition. The traditional Chinese herb Centella asiatica has pharmacological effects, including clearing heat, detoxifying, detumescent and haemostatic effects. Chi [72] and Cai [73] used *C. asiatica* extracts, asiatic acid, and asiaticoside to fabricate MNs, and the results revealed their antibacterial function. Green tea extracts have potent antibacterial effects, which inhibit the growth of gram-negative bacteria (*E. coli*, *Salmonella typhimurium* and *Pseudomonas putida*) and gram-positive strains (*Bacillus subtilis* and *Staphylococcus aureus*) [74]. Carvacrol (CAR) has been fabricated as nanoparticles (NPs) and exhibited sustained antibacterial function at the infection site [75]. Yunan Baiyao, known for its ability to promote platelet adhesion and activate the coagulation system, has been designed as an MN base for rapid haemostasis [33]. Tanshinone IIA, an active ingredient derived from *Salvia miltiorrhiza*, inhibits dysplastic fibroblasts and has been used to treat scars [76].

### 5.2 Delivering-growth factors MNs

Growth factors, such as epidermal growth factor (EGF), transforming growth factor-β (TGF-β), fibroblast growth factor (FGF), vascular endothelial growth factor (VEGF), and platelet-derived growth factor (PDGF), participate in the inflammatory and remodelling phases of wound healing by regulating epithelialisation, collagen deposition, and angiogenesis [77]. Chronic wounds are often accompanied by infiltration of inflammatory cells, down-regulation of growth factors, and up-regulation of proinflammatory factors, resulting in poor wound healing. Topical application of growth factors is used clinically to accelerate healing [78,79]. However, some problems arise with topical applications. Growth factors are easily hydrolysed by proteases in the wound environment due to their instability, thereby diminishing their therapeutic efficacy. MNs loaded with growth factors effectively solve this problem. MNs encapsulated with bFGF in PLGA microspheres have been demonstrated to sustain release for 15 days [80]. Chi et al. developed a temperature-responsive VEGF-releasing chitosan MN for its antibacterial and angiogenic properties [81]. MNs loaded with recombinant human EGF (rhEGF) have been shown to inhibit inflammation and promote angiogenesis, collagen deposition, and tissue regeneration [32,82].

### 5.3 Delivering metal MNs

#### 5.3.1 Metal nanoparticles

Antibiotic resistance is gradually increasing. Metal nanoparticles (NPs) with antimicrobial capacity and strong biocompatibility have aroused attention in the pharmaceutical industry, such as silver [83], zinc oxide [84,85], zinc [86,87], iron [88,89], magnesium hydride [90], and other metal NPs. These metal NPs interfere with mitochondrial function, promote the release of reactive oxygen species (ROS), penetrate the cell membrane to acess DNA, and cause nuclear damage and cell death. To ensure the solubility of metal NPs in polar solvents, stabilising agents, such as citrate, must be added to jacket the metallic nucleation sites [91]. Citrate carries negative ions around the outside, making the uncharged metal NPs negatively charged. However, the bacterial cell wall also carries a negative charge, making surface adhesion difficult between NPs and the cell wall. Du et al. prepared CS-MoS_2_ MNs, and positively charged CS enhanced the adhesion of MoS_2_ to the bacteria to improve antibacterial efficiency [92]. Yang et al. fabricated CS/BSP (*Bletilla striata* polysaccharides) MNs loaded with silver NPs, which showed the potential to promote the healing of infected and susceptible wounds [83]. NPs have poor dispersibility and a small specific surface area in general media, which affects their antimicrobial capacity. Ionic liquids (ILs) refer to molten salts whose cations are organic, including imidazole salts, piperidine salts, pyridine salts and pyrrole salts. ILs, with a loose structure, a small force between anions and cations, and a low melting point can exist stably at room temperature, so they can be used as a dispersion medium for various NPs. ZnO/IL MNs exhibit better antimicrobial properties than single ZnO NPs [84].

#### 5.3.2 Metal peroxides

Metal peroxides produce oxygen and hydrogen peroxide (H_2_O_2_) by chemical reactions (Fenton or Fenton-like reactions). H_2_O_2_ produces highly toxic reactive oxygen species (ROS), such as hydroxyl radicals (OH), which kill cells. The family of metal peroxides encompasses calcium peroxide (CaO_2_), copper peroxide (CaO_2_), silver peroxide (Ag_2_O_2_), zinc peroxide (ZnO_2_) and magnesium peroxide (MgO_2_). Woodhouse et al. generated PVP-MNs loaded with CaO_2_ and polyvinylpyrrolidone. The CaO_2_ within these MNs was hydrolysed to produce ·OH in the wound bed, thereby increasing local tissue oxygenation and enhancing the antibiofilm capacity [93].

#### 5.3.3 Nanozymes

Nanozymes are artificial synthetic enzymes with both unique properties of nanomaterials and catalytic function. The peroxidase- and catalase-like activities of Fe_3_O_4_ NPs were first discovered in 2007 by Professor Xiyun Yan. Since then, NPs with enzymatic activity, called nanozymes, have been extensively studied [94]. Zhu et al. used Fe_3_O_4_ nanozymes to catalyse the production of OH to destroy biofilms in weakly acidic and hydrogen peroxide microenvironments. They fabricated MNs loaded with Fe_3_O_4_ nanozymes and graphene oxide (GO) to play a dual role in antibiofilm and photothermic reactions [95]. Sun et al. utilized Fe_2_C NPs, another iron-based nanozyme, in conjunction with glucose oxidase (GOx), to resolve the peroxidase activity of Fe_2_C NPs constrained by pH and H_2_O_2_, which produced more efficient and stable OH to destroy MRSA biofilms [96]. However, an excessive production of ROS can damage normal cells and cause an excessive inflammation response. Thus, Shan et al. designed space-time-responsive MNs with Cu_2_MoS_4_ dual-nanozyme tips and a polydopamine (PDA) NP base. The excessive ROS produced by Cu_2_MoS_4_ dual-nanozyme tips were neutralized by the antioxidant PDA NPs in the base [97]. This dual-layer design achieved precise microregulation of ROS levels.

### 5.4 Delivering-exosomes MNs

Exosomes are extracellular vesicles ranging from 30 to 150 nm in diameter, which are widespread and distributed in various body fluids (e.g. blood, saliva, urine, and cerebrospinal fluid). Exosomes are involved in paracrine-mediated intercellular communication and interactions [98]. Exosomes are endogenous lipid nanoparticles (LNPs), produced by eukaryotic cells, and encapsulated by the bilayer lipid membrane that carries proteins, lipids and nucleic acids (DNA, miRNA, and mRNA) [99]. However, their limited half-life precludes their sustained presence when administered directly into the wound. GelMA/PEGEDA MNs loaded with endothelial cell-derived exosomes and tazarotene significantly promoted collagen deposition, epithelial regeneration, and angiogenesis [100]. Mesenchymal stem cell (MSC) exosome-encapsulated PVA hydrogels as needle tips and 3M detachable medical tape have been used to regulate the proliferation and migration of fibroblasts, promote tube formation of vascular endothelial cells, and polarise M1 macrophages [101]. M2 macrophage-derived exosome-loaded photosensitive hydrogel MNs promoted the transformation of M1 macrophages into M2 macrophages, and exhibited angiogenic properties [102]. These three approaches have been shown to facilitate diabetic wound healing. Consequently, delivering exosome MNs improved their stability and prolonged the therapeutic wound-healing effect [103].

### 5.5 Delivering stem cell MNs

Stem cells have been widely used in regenerative medicine because they differentiate into functional cells and promote tissue regeneration by secreting cytokines. However, the use of stem cells for skin wound repair is hampered by poor adaptability and limited migration. MNs could provide an ideal microenvironment and directly transfer stem cells into the epidermis, thereby overcoming these limitations. Studies by Lee [104] and Wu [105] respectively fabricated MNs loaded with mesenchymal stem cells (MSCs) and adipose-derived stem cells (ADSCs), which maintained more than 24 % cell viability 90 h after generation of the MSCs-MNs, and the ADSCs-MNs observed a proliferative trend in cell viability after culture 72 h. Both groups demonstrated that the administration of stem cells-loaded MNs improved neovascularisation, collagen deposition, and tissue regeneration.

### 5.6 Delivering-plant MNs

**Fig. 5:**
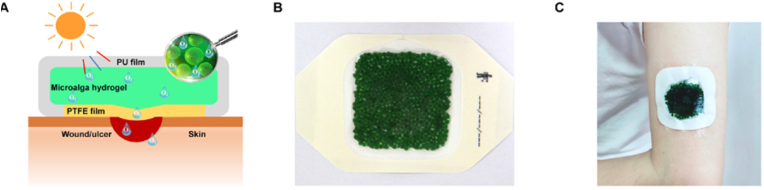
Images of the Chlorella vulgaris-loaded MNs. (A) Schematic illustration of microalga-hydrogel patch preparation through polyurethane film and polytetrafluoroethylene membrane to perform the light response dissolved oxygen release for chronic wound. (B) and (C) Images of CV-loaded MNs on the arm. Reproduced under terms of the CC-BY-NC license [106]. Copyright 2023, Science Advance.

Besides delivering chemical drugs, metals, exosomes and cells, MNs can also be used to deliver plants. *Chlorella vulgaris* (CV) is a small green alga with immunomodulatory and antioxidant capacity that has been widely used in aquaculture and health products. CV has a rapid photosynthetic rate and produces large amounts of dissolved oxygen. Trace elements, such as zinc and magnesium ions in CV, have anti-inflammatory effects. Zhao et al. designed a MN patch dressing with CV-loaded GelMA tips and a PVA base, and *in vitro* experiments demonstrated that CV survived for at least 6 days. CV-loaded MNs continuously infiltrated oxygen to promote angiogenesis, reepithelialisation, and collagen deposition in diabetic wounds (Fig. 5) [106].

## 6 Smart responsive microneedles

The MNs system described to this point release their loaded drugs mainly by diffusion and degradation. When the drug molecule is smaller than the polymer, it is released from the pore of the polymer along a concentration gradient by diffusion. Another way to degrade a drug applies to drugs that cannot directly pass the pores in dense polymers, such as PLGA and PCL, and drug release requires hydrolysis or enzymatic degradation to degrade the polymer. These two modalities are uncontrollable and are difficult to stop once they occur. Smart responsive materials rapidly respond to environmental changes by changing their structure, characteristics, and functions. These MNs provide accurate regulation of the wound bed microenvironment using a stimuli-responsive drug delivery system according to the characteristics of the physical microenvironment (temperature, light, and ultrasound) and the biological/biochemical microenvironment (pH, ROS, glucose, and bacteria).

### 6.1 Thermoresponsive microneedles

The wound undergoes a local inflammatory response resulting in increased skin temperature due to infiltration of inflammatory cells and vasodilation by inflammatory agents, such as histamine. Thermosensitive polymers are class of polymers to modulate their physical states in response to temperature changes, such as poly (N-isopropylacrylamide) (p-NIPAM). They have a specific phase transition temperature called low critical dissolution temperature (LCST) or upper critical dissolution temperature. They could transit between a solvent and gel phase at the LCST [107]. The LCST of p-NIPAM is 32 °C, when the p-NIPAM hydrogel assumes the liquid state, allowing for the incorporation of drugs [108]. Upon reaching the human body temperature, p-NIPAM converts from solution to gel state [109]. When the applied temperature exceeds the volume phase transition temperature (approximately 37 °C), the p-NIPAM hydrogel contracts and then releases the encapsulated drugs [110]. P-NIPAM-based hydrogels have thermoresponsive capacity and are widely used as smart stimulus-responsive materials. Wang [43], Guo [42], and Chi [81] designed p-NIPAM-based MNs for a mouse full-thickness skin wound model, a diabetic foot mouse model, and a rat-infected wound model, respectively.

However, p-NIPAM still exhibits certain deficiencies, including low mechanical strength, limited drug-loading capacity, constrained response rate, and low biodegradability [111], which impede its further advancement. Due to the incomplete biodegradability of p-NIPAM, there may be concerns regarding long-term biotic accumulation.

### 6.2 Light-responsive microneedles

Phototherapy is a non-invasive treatment that uses specific wavelengths of light to treat diseases. The electromagnetic waves most commonly used for light-responsive MNs are near-infrared (NIR) waves. Photothermal treatment (PTT) refers to the use of a photothermal agent (PTA) to convert light into heat energy under external illumination, such as NIR light. Su et al. used IR 780 iodide as a PTA, along with antimicrobial peptide W379 loaded in PVP MNs with surface-sprayed tetradecanol (TD). When irradiated by NIR, IR 780-mediated PTT prompted the dissolution of TD, which released antimicrobial peptides [58]. Yao et al. fabricated a novel MOF MN patch possessing a NIR photothermal response by encapsulating GO [112]. This MN patch controlled the release of nitric oxide (NO) to promote wound healing. Shan et al. constricted NIR-II responsive, glucose-responsive, and dual-enzymatic Au-CMS nanoscale MNs (NSMNs), and experimentally demonstrated that NIR-II induced overheating and kills MRSA [113]. Sun et al. utilized MXene, which encompassed transition metal carbides and nitride material, and had photothermal conversion capacity, and accelerated the release of loaded adenosine under NIR irradiation to promote healing [114]. Zhang et al. designed a detachable NIR-responsive MN loaded with black phosphorus (BP) and haemoglobin at the tip. The exothermic oxidative reaction of BP was triggered by NIR irradiation, raising the skin temperature. The oxygen-blinding capacity of haemoglobin gradually decreased because of the high temperature, which caused the release of oxygen to the wound bed and controllably delivered oxygen to the deep dermis [115].

**Fig. 6:**
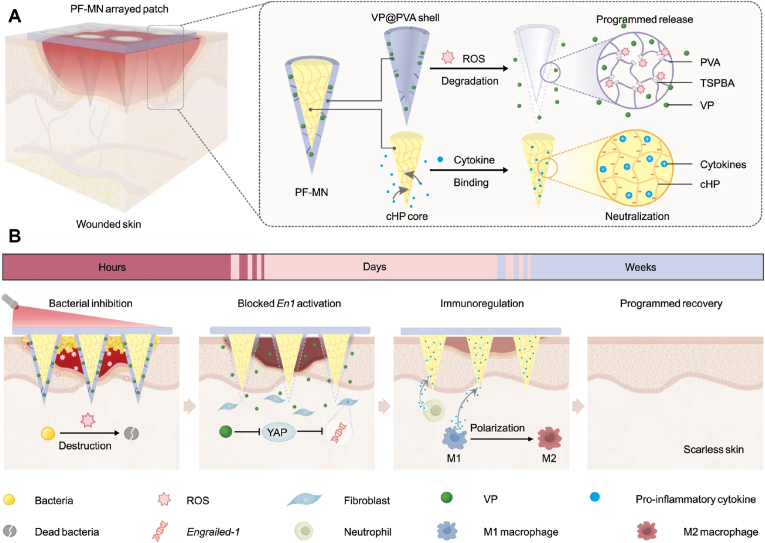
Schematic illustration of the structure of PF-MNs and the programmed regulation process for chronic wound, including elimination of bacterial biofilm via generating ROS, release of VP for scarless wound regeneration, and cytokine neutralization as well as macrophage transformation by cHP core. Reproduced under terms of the CC-BY license [116]. Copyright 2023, Nature Communications.

Photodynamic treatment (PDT) use photosensitizers (PSs) to convert oxygen into ROS under light irradiation at appropriate wavelengths. Porphyrins and their derivatives are the most widely used as PSs. Verteporfin, a second-generation porphyrin PS, has been used to fabricate procedural functional (PF) MNs that produce ROS in antibacterial membranes exposure to NIR light. Verteporfin inhibits scar formation by blocking engrailed-1 activation in fibroblasts [116] (Fig. 6). Wang et al. used etrakis (4-pyridyl) porphyrin as a PS and encapsulated it with sulfobutyl ether-β-cyclodextrin to produce soluble MNs that effectively eradicated biofilm [117]. MOFs can also be designed with photothermal conversion and nanozyme properties, such as multifunctional porphyrin-like metal-centred NPs [118]. Chen et al. designed nanomotors loaded with PS indocyanine green (ICG) and the NO-donor L-arginine, which exerted both photothermal and photodynamic effects under NIR. Singlet oxygen (^1^O_2_) has antioxidant capacity and reacts with L-arginine to produce NO [119]. Yu et al. combined enzymatic hydrolysis, antibiotics, and photothermal reactions to prepare α-amylase-levofloxacin @ PDA NP MNs to synergistically clear biofilms from the wound bed [54]. PDT is also applied in scar treatment. Chen et al. designed a drug delivery MOF and autophagy inhibitor (chloroquine MNs) to solve the limitations of low transdermal efficiency of PSs and cytoprotective lysosomal autophagy [120].

Although MNs based on PTT and PDT have many advantages, they are not without limitations. PTT is difficult to stop once initiated, leading to challenges in controlling local temperature. The excessive heat may result in tissue or cellular damage. PDT requires oxygen molecules to produce ROS, thus its efficacy may be compromised in hypoxic environments. PDT eliminates bacteria by generating ROS. However, chronic wounds inherently have high levels of ROS, and the addition of ROS produced by PDT can lead to an excessive accumulation of ROS that causes tissue damage.

### 6.3 Ultrasound-responsive microneedles

**Fig. 7:**
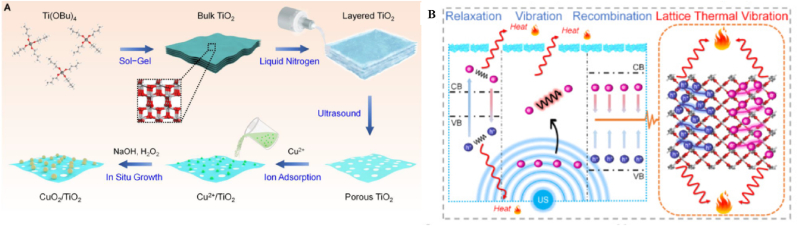
Synthesis of CuO_2_/TiO_2_ integrated MNs. (A) Schematic illustration of the synthetic route of CuO_2_/TiO_2_ nanostructures. (B) Schematic illustration of the sonothermal mechanism of CuO_2_/TiO_2_ under ultrasound. Reproduced with permission [123]. Copyright 2023, Acta Biomaterialia.

Ultrasound is a type of mechanical wave with a frequency higher than 20 kHz. Ultrasound waves act on cells through a cavitation effect, referring to the cavitating bubbles that rapidly expand and rupture after absorbing light energy, producing high temperatures (up to 10,000K) and high pressure (81 MPa). The energy released from this process induces dissociation hydrolysis to produce hydroxyl radicals (·OH) [121]. In the materials field, microbubbles based on cavitation effects are designed as ultrasound-responsive drug carriers. Microbubbles expand under ultrasonic waves and ultimately rupture to release the drug inside [122]. Sonodynamic therapy involves the use of ultrasound to activate an ultrasound sensitizer to generate ROS. Liang et al. prepared CuO_2_/TiO_2_-integrated MNs by using titanium oxide (TiO_2_) as the ultrasound sensitizer, which achieved the bilaterally augmented sono-chemodynamic and sonothermal antibacterial therapy [123] (Fig. 7).

Nevertheless, the mismatch in acoustic impedance between ultrasound-responsive materials and biological tissue may lead to reflections and reduced absorption efficiency of ultrasonic energy, compromising its effectiveness. The short wavelength of ultrasound and the absorption of some energy by tissue, result in a reduced response in deep tissues. Moreover, because the high-intensity ultrasound can cause damage to human tissue, the intensity of ultrasound and the exposure duration need to be strictly controlled.

### 6.4 pH-responsive microneedles

**Fig. 8:**
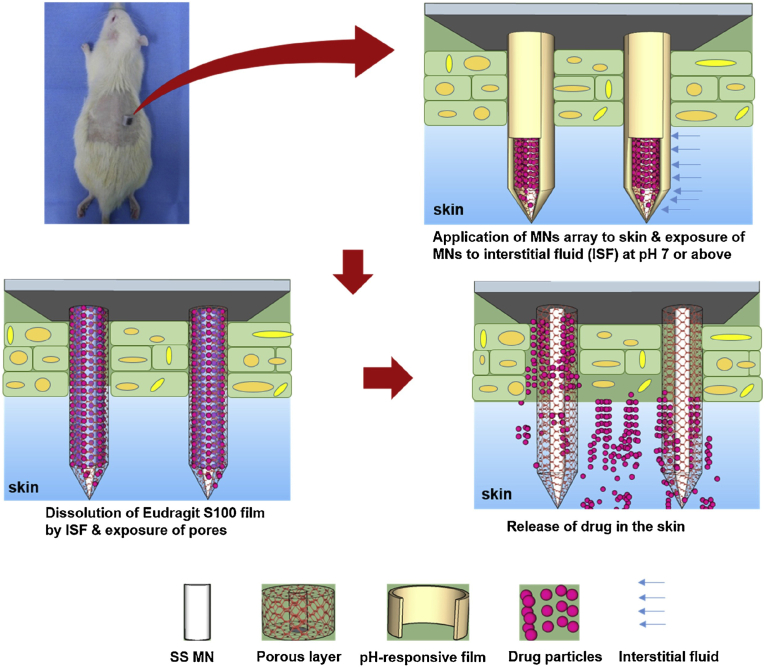
Wound pH-dependent release system schematic illustration. Reproduced with permission [126]. Copyright 2021, Sensor and Actuators B: Chemical.

Normal skin pH ranges from 4 to 6.5. Acute wounds exhibit an acidic milieu with a pH between 5 and 6, while chronic wounds are characterized by an alkaline environment with a pH spanning 7 to 9 because of infection. There is a correlation between the alkalinity of chronic wounds and their severity [124,125]. Acidic environments are suitable for angiogenesis and epithelial formation, and help release oxygen and maintain resident commensal bacteria. Increasing pH promotes the conversion of a chronic wound into an acute wound, thereby promoting wound healing. Eudragit S100 is a pH-sensitive polymer that is soluble in an alkaline environment rather than an acidic environment. Ullah et al. coated Eudragit S100 on the surface of MNs and showed *in vivo* (rat abrasion wound model) and *in vitro* (phosphate-buffered saline and isolated porcine skin) wound pH-sensitive drug release, where the drug release rate increased under the wound pH condition (pH 7.5) [126] (Fig. 8). The drawback of pH-responsive MNs lie on the relatively narrow pH window for promoting wound healing. To achieve precise control of drug release, higher demands are placed on the materials and structural design.

### 6.5 Bacterial-responsive microneedles

**Fig. 9:**
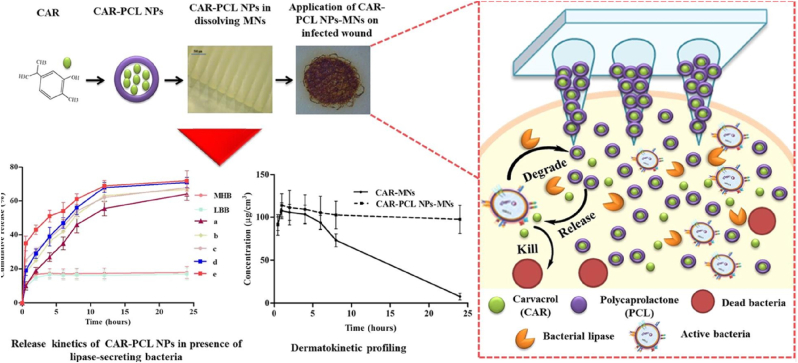
Schematic illustration of the synthetic of CAR-PCL NPs-MNs. Reproduced under terms of the CC-BY license [127]. Copyright 2020, European Journal of Pharmaceutics and Biopharmaceutics.

Chronic wounds are often associated with bacterial infections. Mir [127] (Fig. 9) and Permana [27] utilized PCL NPs as bacterial-responsive materials. PCL NPs degrade in the presence of lipase secreted by bacteria such that the loaded drugs are released under conditions of bacterial growth. Mir et al. loaded CAR, an antibiotic against resistant bacteria, into PCL NPs and incorporated them into PVA/PVP MNs [75]. Permena et al. fabricated PLGA/PCL MNs by loading DOX into CS-coated PCL NPs [27]. Both studies showed that the MNs eliminated the biofilms in *ex vivo* porcine skin studies. Xu et al. exploited gelatinase as another bacterial-responsive enzyme produced by bacteria. They loaded gelatinase-sensitive gelatine NPs and chloramphenicol to achieve responsive release of chloramphenicol, which played a bactericidal role. The *in vitro* antibacterial experiments indicated that chloramphenicol-loaded gelatine NP MNs were superior to free chloramphenicol solutions in killing bacteria [56].

Bacterial composition and concentration of human wounds are more complex compared to animal models. The bacterial-responsive MNs may not be high selectivity in recognizing specific bacterial species and respond to multiple bacteria, leading to misdiagnosis or mistreatment. The sensitivity of MNs may be insufficient for detecting low concentrations of bacteria, particularly in the early stage of disease. Additionally, MNs may exhibit differential responses to various bacterial forms, such as spores, dormant cells, influencing the accuracy of the results.

### 6.6 Glucose-responsive microneedles

High glucose levels impede diabetic wound healing. Therefore, glucose-responsive MNs are designed to control drug release. The most common glucose-responsive substances include a phenylboronic-based polymer (P-PBA), GOx and concanavalin A (Con A). PBA specifically binds to diols on glucose to form reversible phenylboronate ester bonds. GOx specifically catalyses β-D-glucose and oxygen to produce gluconic acid and H_2_O_2_ under aerobic conditions, leading to a decrease in pH and glucose levels. Con A is a tetrameric globulin, each subunit of which contains a sugar-binding site that specifically binds to α-mannose and α-glucose [128]. PBA and Con A cannot reduce glucose levels.

**Fig. 10:**
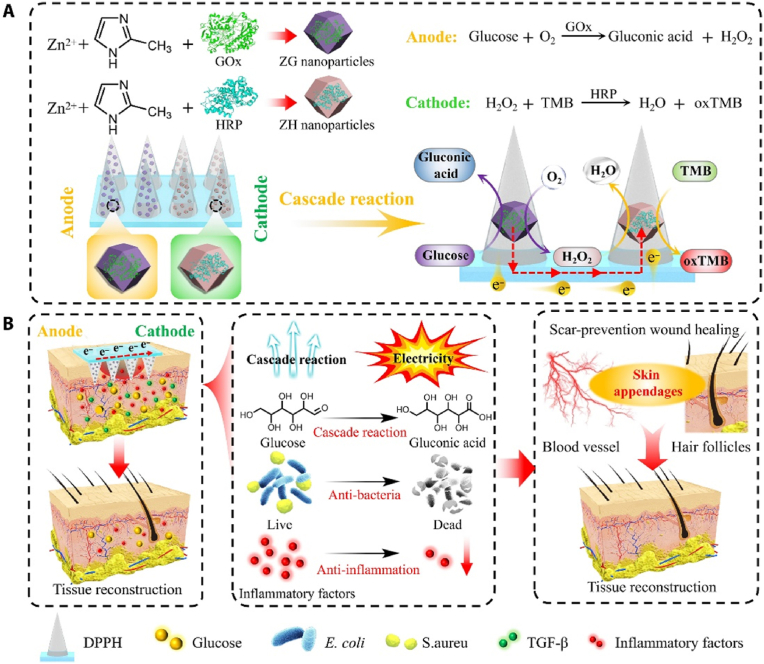
Schematic diagram of self-powered MN patch. (A) Schematic of the enzyme cascade reaction generated by the self-powered MN patch. (B) Schematic of the self-powered enzyme-linked MN patch for accelerating diabetic wound healing. Reproduced under terms of the CC-BY-NC license [131]. Copyright 2023, Science Advances.

Guo et al. achieved glucose responsiveness by using 4-(2-acrylamide ethyl carbamoyl)-3-fluorophenylboronic acid (AFPBA). AFPBA-MNs release insulin in response to changes in glucose concentration, thereby accelerating diabetic wound healing, reducing the inflammatory response, controlling blood glucose, and promoting tissue regeneration [129]. Yu et al. prepared MNs with glucose-responsive vesicles (GRVs) loaded with insulin and GOx, and the GRVs were self-assembled from hypoxia-sensitive hyaluronic acid (HS-HA) and 2-nitroimidazole (NI). Glucose enzymatic oxidation in diabetic hyperglycaemia causes a locally hypoxic microenvironment. A hypoxic environment promotes the reduction of HS-HA and hydrophobic NI to reduce hydrophilic NI, which dissociates the vesicles thus promoting the release of insulin [130]. Zhang et al. designed a self-powered enzyme-linked MN patch with GO and horseradish peroxidase (HRP) loaded in the anode and cathode respectively, which rapidly responded to diabetic wounds and released stable microcurrents to prevent scar formation (Fig. 10) [131].

Glucose-responsive MNs suffer from a problem of delayed response time, falling to immediately react to rapid changes in blood glucose levels, which may cause unstable glucose control. Glucose-responsive materials may cross-react with other carbohydrates or reducing substances, resulting in erroneous signal output and unintended drug release.

## 7 Conclusions and perspectives

With the aging of the population and the increasing frequency of chronic diseases, the incidence of difficult wound healing has been increasing annually, which has imposed a huge economic burden on individuals and society. MNs, as a new generation of wound dressings, exhibit better drug delivery than traditional dressings, such as creams and hydrogels, and are also painless. In wound treatment, MNs achieve local penetration though necrotic tissue, eschar, biofilm, and epidermis into deep tissues. This approach circumvents the issues associated with drug dilution and degradation, thereby significantly improving the efficiency of drug delivery. MN patches have great potential as wound dressings. Smart responsive MNs have been manufactured according to the different physicochemical/biological characteristics of the wound microenvironment to achieve refined and responsive regulation. Different biologically, biochemically, and physically responsive materials have also been integrated.

However, MNs are not yet ready for clinical use. First, the actual effects of MNs lack clinical evidence. According to the literature, most MNs have been tested in rodent models, such as mice and rabbits, which differ in skin regeneration and scar formation mechanisms compared to humans. Second, MNs deliver a greater variety of drugs. In addition to the drugs mentioned in this review, the microbiota, which are widespread, can be genetically engineered to secrete antimicrobial substances and growth factors that promote wound healing. Lu et al. genetically engineered *L. lactis* to continuously express VEGF and induce the polarization of lactic acid-induced macrophages [132]. Third, MNs must have spatially and temporally flexible release capabilities. Wound healing is a dynamic and complex process that requires different factors to intervene in different phases. For example, the early haemostasis phase requires procoagulants, the inflammatory phase requires antibacterial drugs, the proliferative phase requires growth factors to promote proliferation, and the remodelling phase requires inhibition of fibroblast hyperproliferation. Most current MNs deliver homogeneous substances and cannot be released in different spaces of the wound bed to support various cell functions. In the future, one potential direction is that spatiotemporal release can be achieved based on material degradation capabilities and MN types (bilayer or multilayer MNs). Another potential direction is the development of a multi-responsive smart microneedle system designed to facilitate smart delivery in response to diverse stimuli or a combination of pharmaceutical agents. Fourth, the MN fabrication process is complex, and the materials are expensive and not suitable for industrial production. How to fabricate cheap, safe, and effective MNs is the first issue to be considered before clinical application. Last but not least, the biosafety of MNs must be taken into account. Although most studies related to MNs have been performed with cytotoxicity tests such as MTT and CCK8, adequate and comprehensive foundational researches are still needed. MNs require the biocompatibility investigations prior to clinic, including hemolysis test, acute/chronic system toxicity, irritation tests, implantation tests, intradermal reactivity tests, and carcinogenicity tests.

In conclusion, MNs have emerged as a new approach to wound treatment and transdermal drug delivery. Smart responsive MNs designed for different biochemical and physical environments provide personalised treatments for patients. In response to limitations, physicians, scientists and engineers should collaborate to fabricate more cost-effective materials, improve capacity, and establish a foundation for future clinical applications.

## Funding

This work was financially supported by the National key research and development project (2022YFC2403100, 2022YFC2403104), the 10.13039/501100001809National Natural Science Foundation of China (81772069, 81801911), and the medical and health science and technology project of Zhejiang province (2020KY786).

## CRediT authorship contribution statement

**Meixuan Liu:** Writing – review & editing, Writing – original draft, Formal analysis, Data curation. **Jing Jiang:** Writing – review & editing, Visualization, Data curation. **Yiran Wang:** Writing – review & editing, Writing – original draft. **Huan Liu:** Formal analysis, Data curation. **Yiping Lu:** Writing – original draft, Data curation. **Xingang Wang:** Conceptualization.

## Declaration of competing interest

The authors declare that they have no known competing financial interests or personal relationships that could have appeared to influence the work reported in this paper.

## Data Availability

No data was used for the research described in the article.
